# Sandwich bone graft for vertical augmentation of the posterior maxillary region: a case report with 9-year follow-up

**DOI:** 10.1186/s40729-017-0063-9

**Published:** 2017-05-19

**Authors:** Kenko Tanaka, Irena Sailer, Yoshihiro Kataoka, Shinnosuke Nogami, Tetsu Takahashi

**Affiliations:** 1 Division of Oral and Maxillofacial Surgery, Department of Oral Medicine and Surgery, Tohoku University Graduate School of Dentistry, 4-1 Seiryo-machi, Aoba-ku, Sendai, 980-8575 Miyagi Japan; 2 Division of Fixed Prosthodontics and Biomaterials Clinic of Dental Medicine, University of Geneva, 19 rue Barthélemy-Menn, CH-1205 Geneva, Switzerland

**Keywords:** Bone graft, Long-term follow-up, Interpositional bone graft, Sandwich graft

## Abstract

The loss of teeth followed by bone resorption often lead to defects in the alveolar ridge, making installation of dental implants difficult. Correction of such bone defects, especially lack of height of the ridge, is a difficult problem for all dental surgeons. This report describes the outcome of treatment after alveolar ridge augmentation in the atrophic posterior maxillary region via segmental sandwich osteotomy combined with placement of an interpositional autograft prior to placement of endosseous implants. The technique was successfully used to treat a deficiency in the vertical dimension of the posterior maxillary region. Six months after graft surgery, two implants were successfully placed in accordance with the original treatment protocol, and they survived for 9 years of follow-up.

## Background

Osseointegrated implants for the replacement of missing teeth have recently become a routine treatment option [1, 2]. However, any tooth loss may be followed by extensive resorption of the alveolar ridge, which usually makes implant placement difficult or impossible because of the lack of bone volume. There are a variety of defect situations with increasing complexity, ranging from fenestrations, to dehiscences, to both horizontal and vertical deficiencies, while combinations of these also occur. Ridge augmentation techniques are available to effectively and predictably increase the width of the alveolar ridge in horizontal deficiencies. If vertical deficiencies are present, including in combination, the predictability of the techniques is usually substantially lower [3]. A significant bone defect is an anatomical limitation that can be overcome using different surgical techniques, including vertically guided bone regeneration. Several techniques are currently employed, using some combination of autologous bone or biomaterials, various vertical guided bone regeneration (GBR) procedures [4, 5], alveolar distraction osteogenesis [6], titanium mesh [7], and onlay bone graft [8].

While the vertical augmentation of the bone has been demonstrated with different techniques, the number of complications and failures of the augmentation procedure is still too high to recommend a widespread use of such procedures [9–11]. In addition, vertical augmentation procedures on compromised alveolar ridges are technically sensitive and might cause significant postoperative morbidity and complications, such as severe postoperative pain, swelling, or graft resorption. Furthermore, augmentation procedures always increase cost, morbidity, and treatment time [12].

Recently, rough-surface implants made with new technology have demonstrated better mechanical and biologic characteristics than traditional machined-surface implants. Several clinical studies have demonstrated high success rates and predictable clinical outcomes for placement of short implants. Short implants have been proposed as an alternative to avoid the problems associated with vertical augmentation [12–14]. Still, there is a need for more clinical studies to support this recent concept.

In the literature, the technique of segmental osteotomy accompanied by interpositional grafting has been reported as a practical and predictable procedure with a low incidence of complications and a high probability of success [15–19]. This approach leaves the soft tissue on the oral side of the midcrestal incision attached to the crestal bone segment. Various studies have shown that alveolar osteotomy associated with interpositional grafting may be an effective alternative to other surgical techniques for increasing vertical bone height in the posterior maxilla and mandible [15–19]. The technique is based on interposing a bone graft between osteotomized bony segments, which act as a “sandwich,” offering good vasculature to both the segment and the graft and resulting in less bone resorption compared to the methods described before [15–19].

This case report describes clinical treatment using segmental osteotomy with interpositional bone grafting to rehabilitate the alveolar ridge in the posterior region of the maxilla with 9 years of follow-up.

## Case presentation

**Fig. 1:**
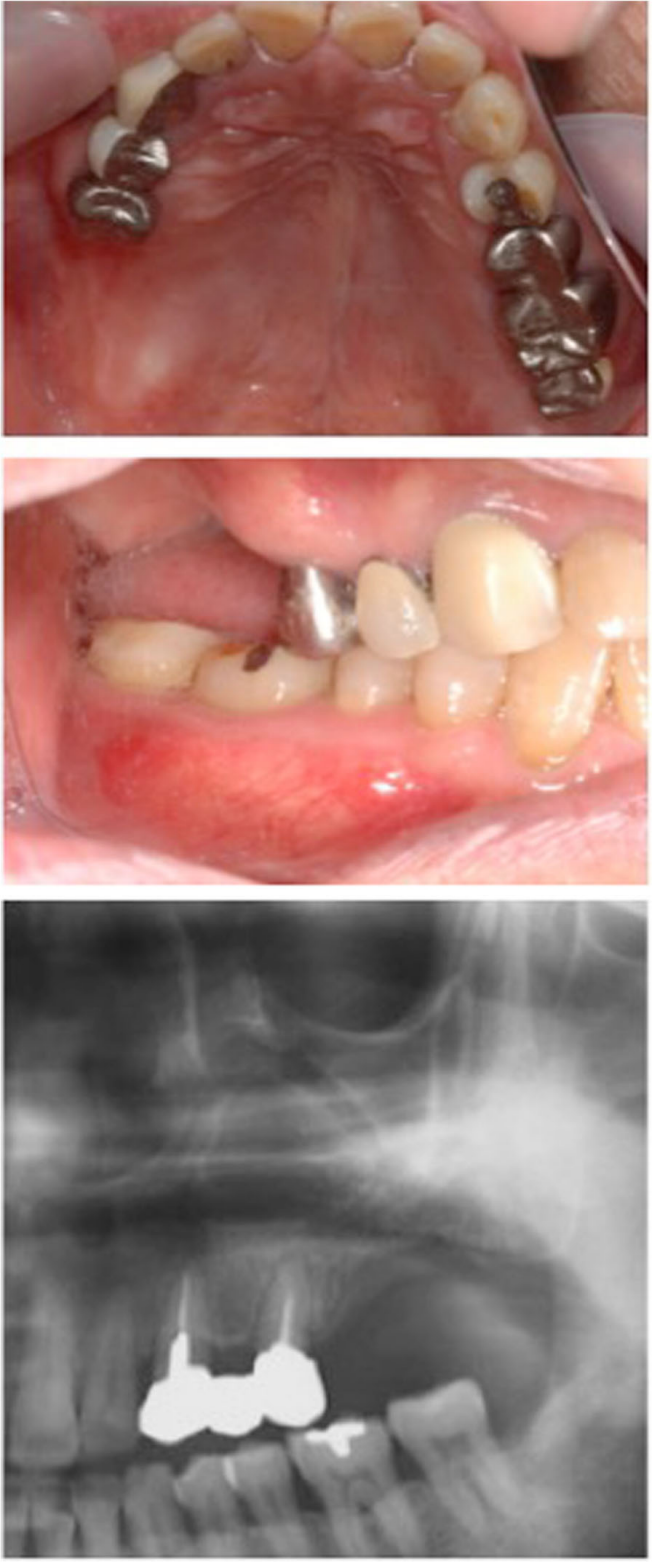
Preoperative intraoral photograph and radiograph

**Fig. 2:**
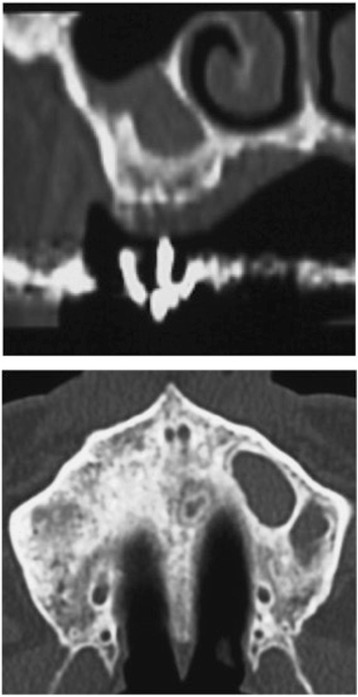
Septa and thickened sinus membrane within maxillary sinus

A 67-year-old male patient sought implant rehabilitation for the purposes of restoration of occlusal support and assistance with chewing difficulties. Clinical and radiological examinations revealed that teeth were absent 26–27. The clearance from the alveolar ridge to the opposing teeth was 20 mm (Fig. 1). A CT scan showed that the distance from the reabsorbed ridge to the floor of the maxillary sinus was approximately 26: 6.1 mm and 27: 7.5 mm, and the width of clearance was approximately 8 mm. The alveolar bone defect in this case was the loss of ridge height with normal ridge width, class II according to the Seibert classification [20]. Additionally, septa and a thickened sinus membrane were evident within the maxillary sinus (Fig. 2).

As a preoperative diagnosis, it was determined that the septa and a thickened sinus membrane meant that sinus lift augmentation was difficult, and bone augmentation to the crown side was required, but the morphology of the alveolar ridge had been well maintained. The treatment options included using short implants, but evidence on their long-term outcome was still limited at that time.

It was determined that the best treatment involved segmental osteotomy and placement of an interpositional graft using the bone removed from the ramus of the mandible to restore the posterior maxillary alveolar ridge, prior to placement of dental implants.

**Fig. 3:**
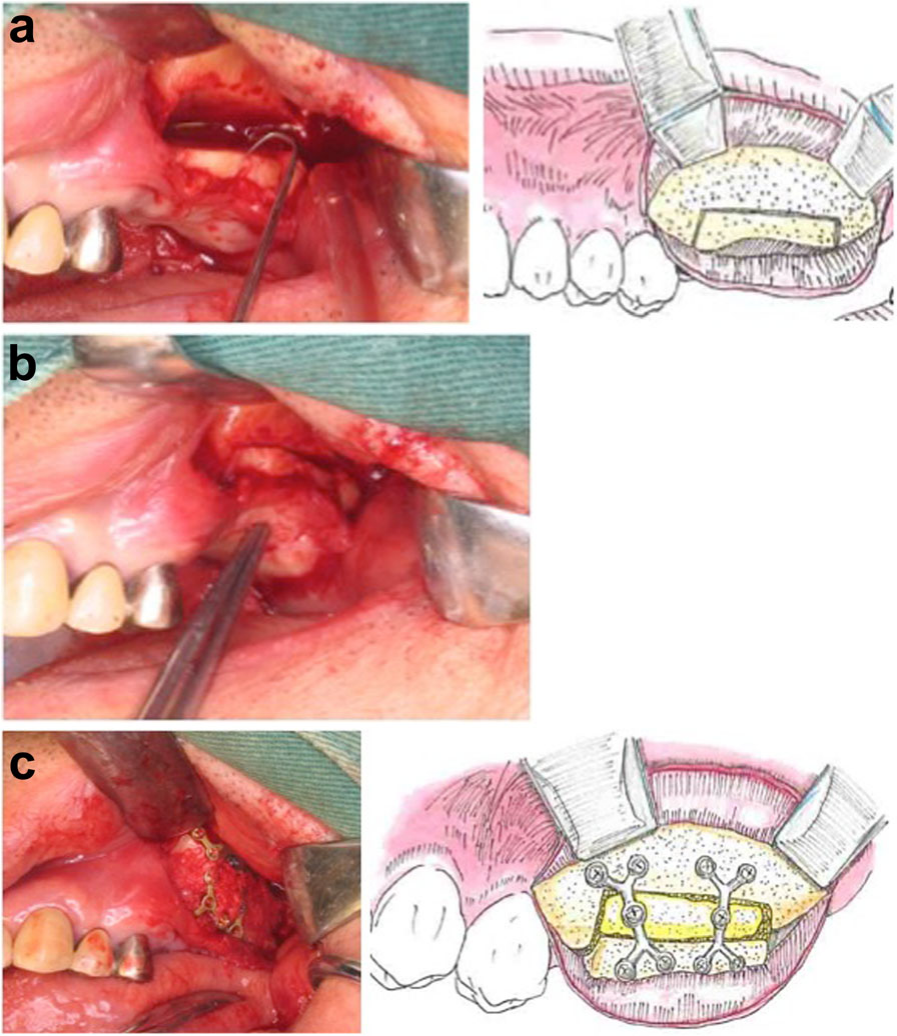
**a** A paracrestal incision was made on the buccal side, and horizontal and vertical osteotomies were made with a piezo-electric device. **b** Placement of the ramus bone block as an interpositional graft. **c** Ramus bone graft fixed

The operative procedure was performed after the induction of general anesthesia using a 1/160,000 xylocaine solution with epinephrine 1:100,000. A linear incision was made 3 mm above the mucogingival junction. The mucoperiosteum was detached, and the vertical and horizontal osteotomies were prepared using micro-saws. Chisels were used to finalize the osteotomies and to mobilize the bony segment. Care was taken not to damage the palatal mucosa. The surgery proceed to the removal of a bone graft block (17 × 10 × 4 mm) from the ramus of the left mandible and the adaptation thereof to the recipient site with the cortical portion facing the vestibular side (Fig. 3). The device formed by the mobilized bone segment and the interposed bone graft block was fixed using WY-type microplates and screws (Stryker Japan, Tokyo, Japan). Crushed autologous bone was applied to the region of the graft (Fig. 3). The procedure was finalized using a running stitch for closure with 5-0 nylon catgut.

**Fig. 4:**
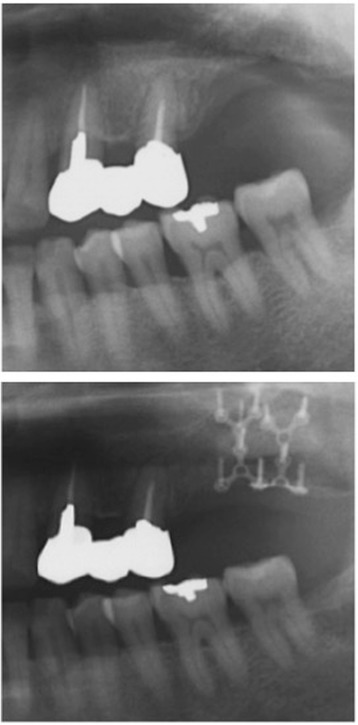
Preoperative and postoperative radiograph

**Fig. 5:**
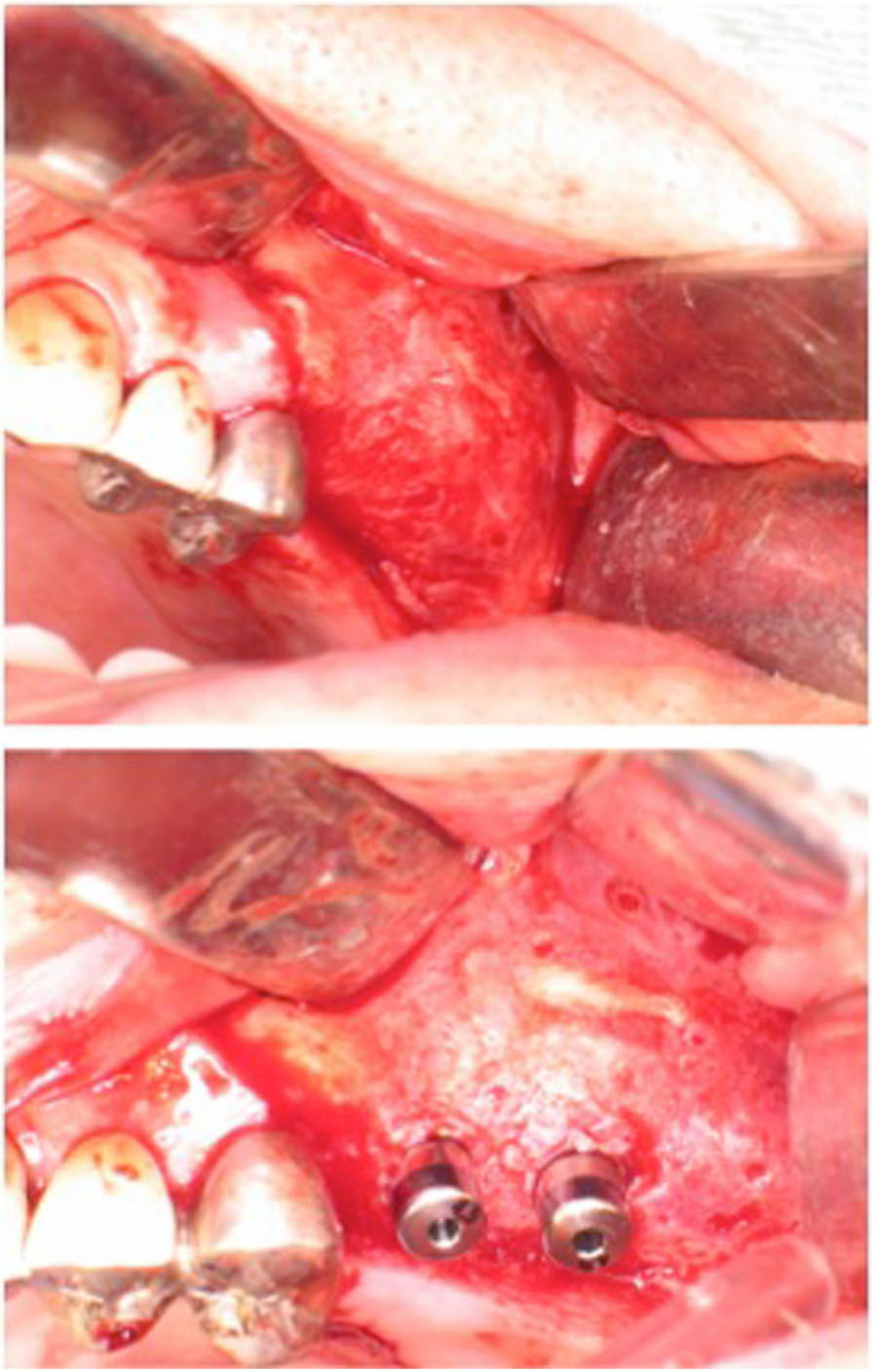
Plate removal and insertion of two implants 6 months after grafting

**Fig. 6:**
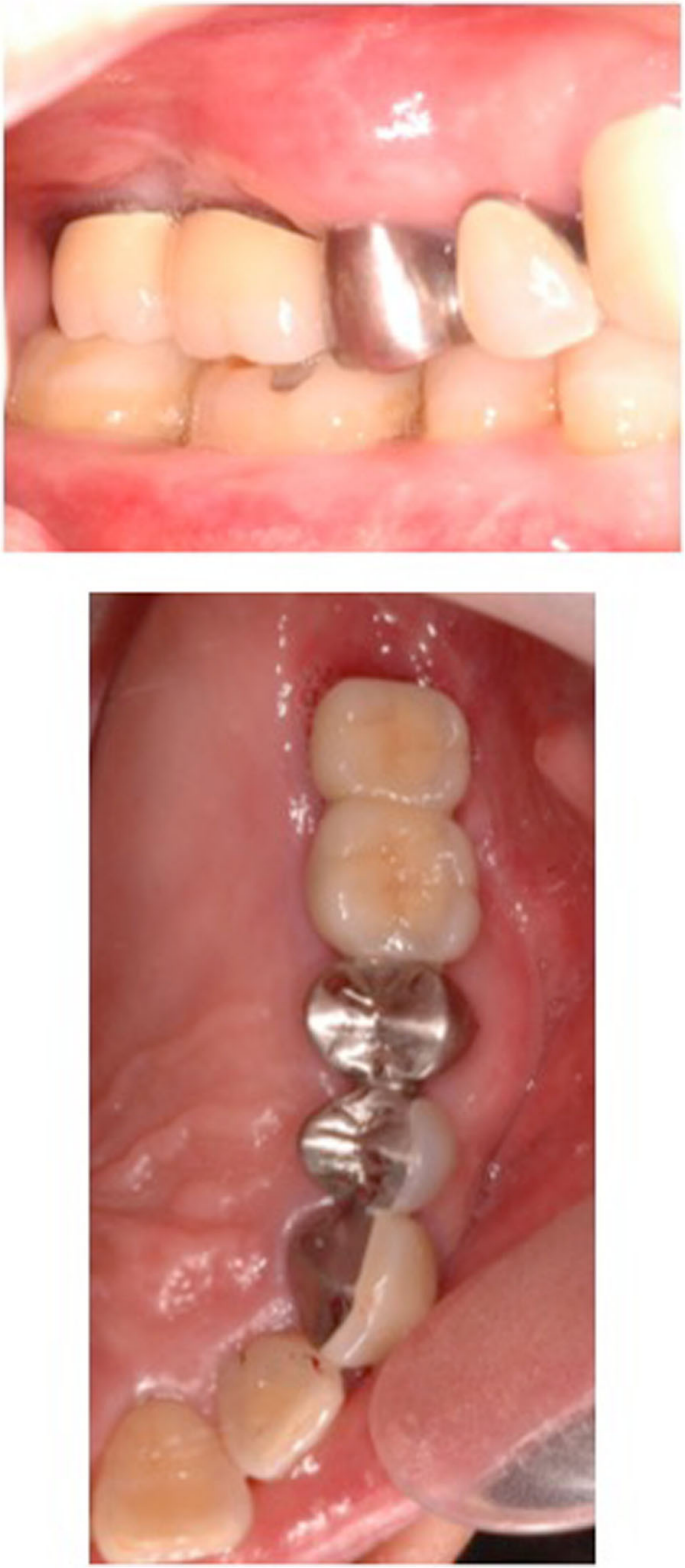
Application of final fixed prosthesis

**Fig. 7:**
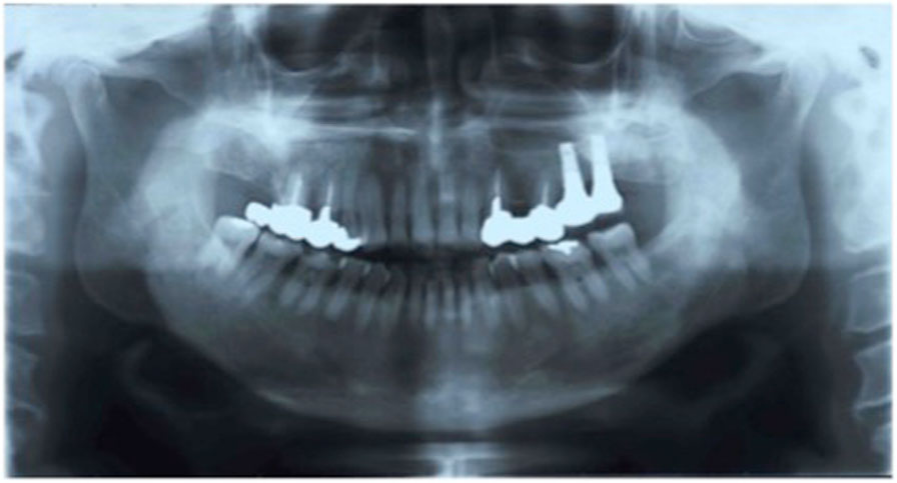
Nine-year follow-up radiograph of the implants

Six months after surgery, radiological examinations were carried out and the patient underwent implant placement (Fig. 4). The postoperative bone height had increased to 10.1 mm at position 26 and 12.9 mm at position 27 compared with the preoperative heights of 6.1 and 7.5 mm, respectively. Postoperative clearance was reduced by 11 mm compared with the preoperative clearance. Careful separation of the mucoperiosteum revealed that the fixation device was in the right place, the interpositional bone graft had been incorporated, and gains in the height and thickness of the alveolar ridge had been achieved. The fixation system was removed, and two dental implants (4.5 × 11 mm) (Astra Tech AB, Mölndal, Sweden) were placed in accordance with the original treatment protocol under the relevant surgical guidance (Fig. 5). Three months after implant surgery, the temporary prosthesis was fixed, and after a further 3 months, the final prosthesis was fixed (Fig. 6). The postoperative course was uneventful for 9 years after surgery (Fig. 7).

## Discussion

This paper reports on a segmental osteotomy procedure with an interpositional graft in the posterior maxillary region with 9 years of follow-up.

The techniques used to overcome a lack of alveolar bone height rely on the placement supplemented by various vertical guided bone regeneration (GBR) procedures [4, 5] and the use of alveolar distraction osteogenesis [6], titanium mesh [7], or onlay bone graft [8]. Gains in ridge height of between 3.6 and 9.2 mm depending on the materials used have been reported, and these were associated with 5-year implant survival rates of 97 to 100%, depending on the method employed [3]. On the other hand, it has also been reported the number of complications (e.g., flap dehiscence, barrier exposure) and failures of the augmentation procedure (e.g. infection, graft bone necrosis) [3–8]. Additionally, the biomaterials used as substitutes for the bone require a longer healing time than autologous bone because the substitutes in general are not osteoinductive [3].

Although a certain amount of slow appositional bone growth from the bony walls into the defect is observed, this growth depends on the growth of new blood vessels between each particle. In the alveolar crest, it spontaneously stops at a distance of few millimeters above the defect bone wall. The more distant particles instead heal within fibrous tissue to form a scar. This is expected to have a negative effect on the long-term survival of the restoration [3].

The use of short implants is another possibility when alveolar bone height is inadequate for regular implants. The use of such implants can reduce treatment time, cost, and postoperative morbidity compared to bone augmentation procedures. The first EAO consensus conference (2006) had defined short implants as a device with a design intrabony length of 8 mm or less [21] and had demonstrated high success rates and predictable clinical outcomes for placement of short implants [12–14], but there were still controversies regarding the long-term consequences of peri-implant bone loss around short implants and its impact on the long-term implant success rate at that time.

In this case, the alveolar ridge was Seibert class II, and septa and a thickened sinus membrane were evident within the maxillary sinus. Sinus floor elevation was limited because of the condition of the floor morphology, the presence of septa, and the thickness of sinus floor membrane [22, 23]. Considering these issues, we selected the interpositional bone graft technique using autologous bone in preference to short implants or the use of a biomaterial.

The inlay bone graft technique, first described by Schettler and Holtermann in 1977 [15] which presented the reconstruction of a severely atrophic edentulous mandible, has great potential for bone graft incorporation. The technique is relatively simple and provides satisfactory results both in terms of surgical success and predictability [15–19]. The technique is predicable because the four walls of the graft are in contact with live tissue, increasing vascularization and reducing resorption [17]. A box-style gap opens between the segments, which borders on an open bone marrow cavity on two sides. This space offers excellent conditions for vascularization of the graft and bone healing. Thus, a temporary prosthesis can be used in the early postoperative period. Since that first report, several reports on research outcomes, technological progress, and the good results obtained with this technique have been published. This technique is now regarded as a good way to correct vertical deficiencies prior to placement of dental implants [15–19].

On the other hand, alveolar augmentation depends on the operator’s experience and is technically sensitive [3]. The most common difficulty is how to manage the soft tissues to preserve the blood supply to the cranial segment; releasing incisions make tension-free closure possible so that the segment does not move palatally.

Nevertheless, in this case, the procedure was carried out successfully, and two regular implants were successfully placed in the alveolar ridge after its enhancement with an autologous bone graft. Those implants survived over 9 years of follow-up.

## Conclusions

We described in the present case a vertical lack of the bone from the alveolar ridge to the opposing teeth, the short distance from the reabsorbed ridge to the floor of the maxillary sinus, and the presence of septa and a thickened sinus membrane within the maxillary sinus. A sandwich bone graft was successfully applied and followed up in the long term. The resulting gains in ridge height and increased thickness of the alveolar ridge appear to have been sufficient for effective placement of the implants, given that these implants have been maintained for 9 years since surgery.
